# Supplementary material for: Stage-Dependent Fibrotic Gene Profiling of WISP1-Mediated Fibrogenesis in Human Fibroblasts
Source: Cells. 2024 Dec 5;13(23):2005. doi: 10.3390/cells13232005 (PMC11640464; doi:10.3390/cells13232005)
Supplement: Supplementary file 1 [file cells-13-02005-s001.zip › 2. Cells_Supplementary figures_03Dec24.pdf]

Supplementary Materials

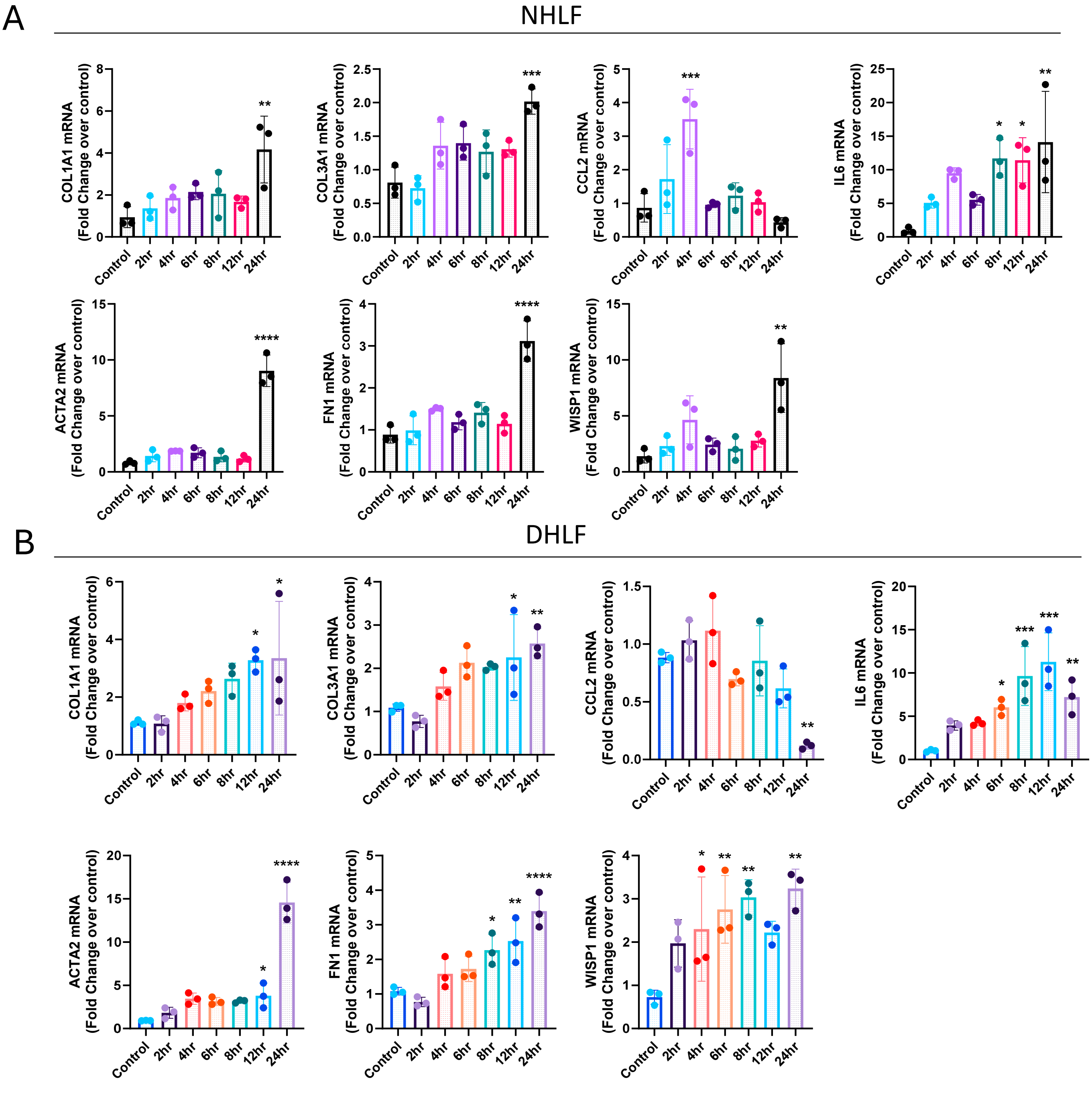

Figure S1: TGFβ time-dependently increases WISP1 expression. Stimulation with TGFβ1 (1ng/ml) yielded a time-dependent increase in profibrotic genes such as Col1A1, Col3A1, ACTA2, FN1 etc. in both **(A)** NHLF and **(B)** DHLF. Statistical analysis was performed using One-way ANOVA with Tukey's Post-hoc analysis vs vehicle control for respective donors. * denotes $p< 0.5$, ** denotes $p< 0.01$, *** denotes $p< 0.001$, **** denotes $p< 0.0001$ versus the vehicle control, as shown.

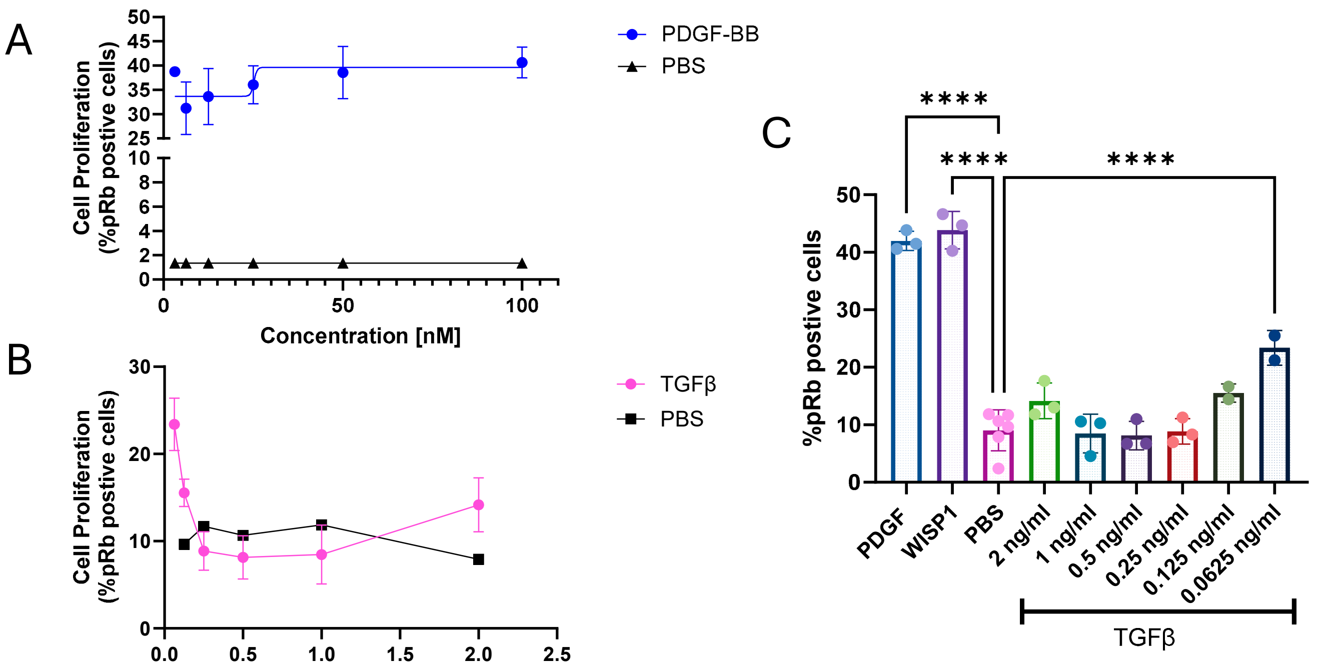

Figure S2: Effect of TGFβ and PDGF-BB on fibroblast cell proliferation. **(A)** PDGF-BB stimulation significantly increases ($p<0.0001$via One-way ANOVA) cell proliferation in a dose-dependent manner. **(B)** TGFβ1 had no significant effect on % pRb positive cells at higher concentration. **(C)** However, lower concentration of TGFβ1 (0.0625ng/ml) significantly increased ($p<0.0001$ via One-way ANOVA) cell-proliferation as compared to control. Three independent experiments (*n*=3) were performed in triplicates. Statistical analysis was performed using One-way ANOVA with Tukey's post-hoc analysis versus the vehicle-control condition and significance denoted as *p<0.5, **p<0.01, ***p<0.001, ****p<0.0001.

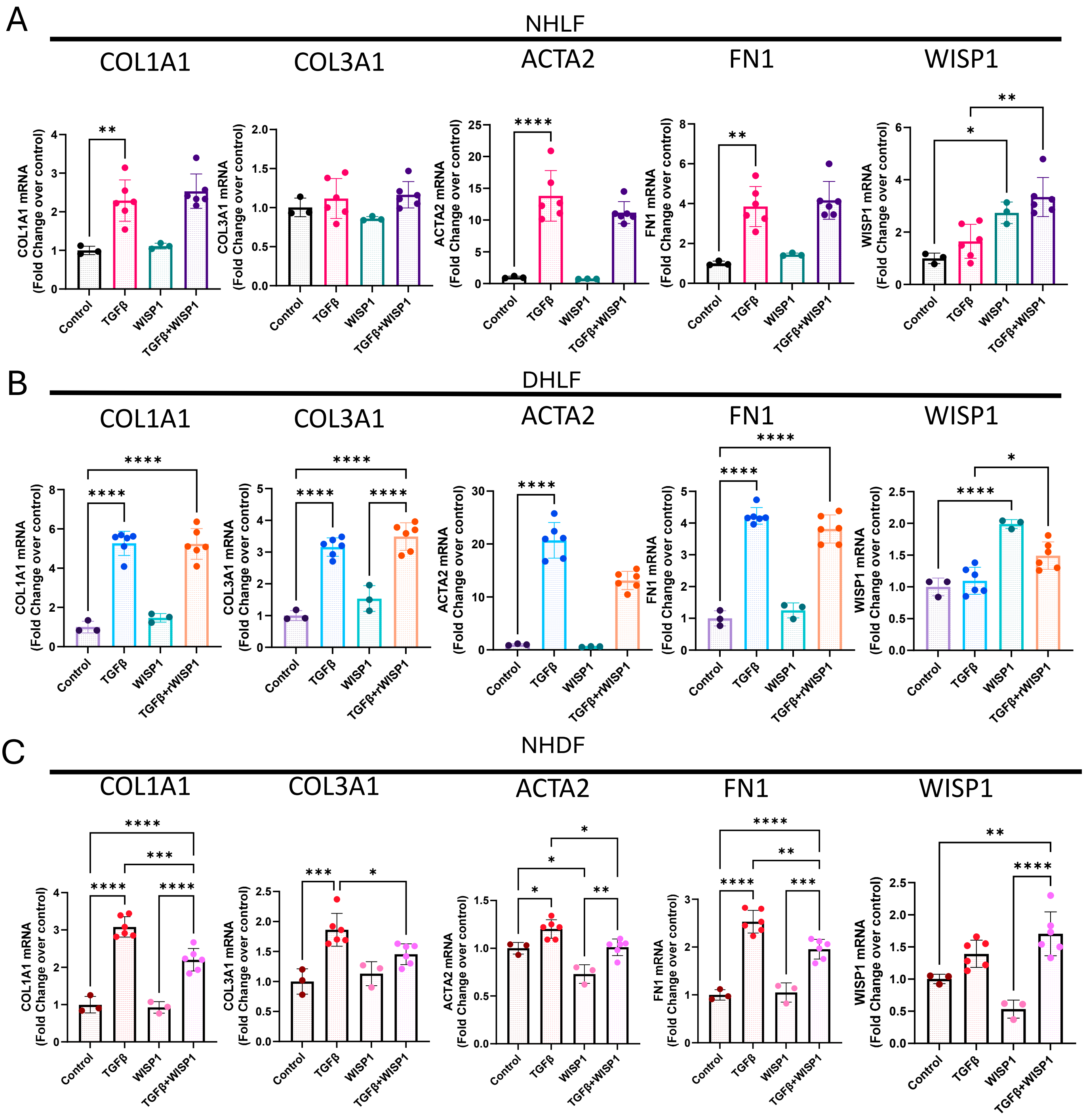

Figure S3: Recombinant WISP1 + TGFβ gene expression analysis.

Primary human fibroblasts were stimulated with TGFβ (1ng/ml) for 24 hr for initiation, followed by WISP1 (1000nM) for another 24hr as per the experimental layout. No significant changes in the profibrotic gene expression, such as *COL1A1*, *COLl3A1*, *ACTA2* and *FN1* were observed in (A) normal human lung, (B) IPF-diseased lung fibroblasts and (C) dermal fibroblast. Statistical analysis was performed using One-way ANOVA with Tukey's Post-hoc analysis vs vehicle control for respective donors. * denotes $p< 0.5$, ** denotes $p< 0.01$, *** denotes $p< 0.001$, **** denotes $p< 0.0001$ versus the unstimulated control, for three biological replicates (n=3).

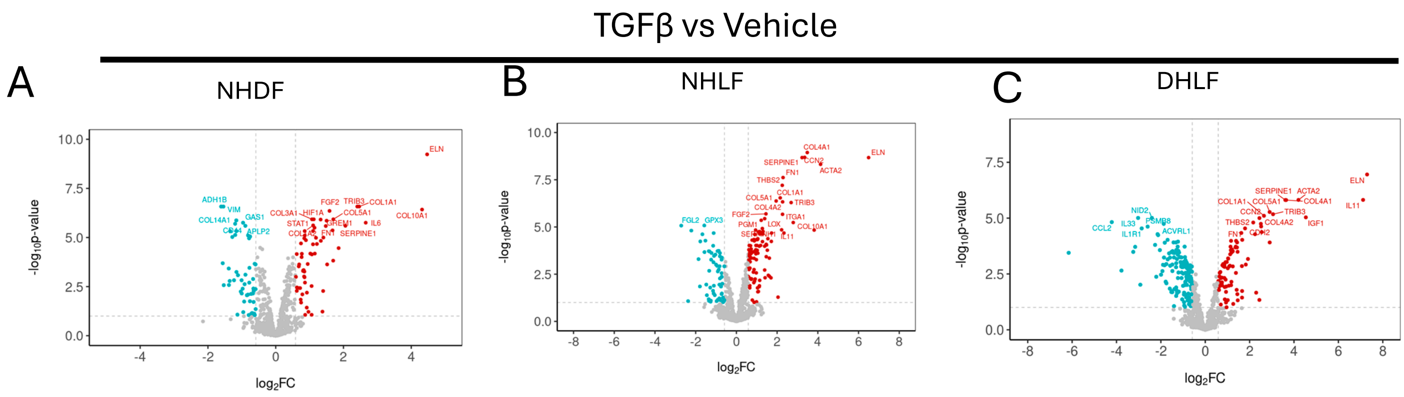

Figure S4: NanoString TGFβ gene signature in lung and dermal fibroblast. The volcano plot illustrates fibroblast genes upregulated (red) and downregulated (blue) upon stimulation with TGFβ **(A)** NHDF, **(B)** NHLF and **(C)** IPF-DHLF as compared to control. The plots represent statistically significant gene with |FC| > 1.5 and Adj Pval < 0.1 are plotted with the x-axis representing $\log_2$Fold change (FC) versus the y-axis $-\log_{10}$ p-value.

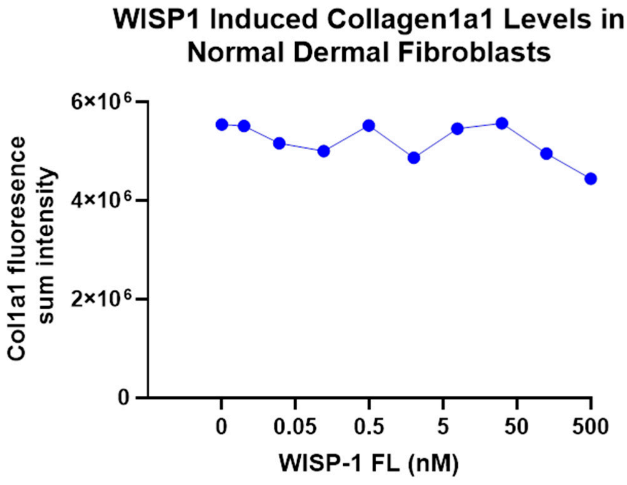

Figure S5: Effect of WISP1 on Collagen levels. Treatment of NHLF with increasing concentration of WISP1 had no significant effect on Col1A1 protein levels as assessed via immunofluorescence.
